# Supplementary material for: The neutrophil to lymphocyte ratio associates with markers of Alzheimer’s disease pathology in cognitively unimpaired elderly people
Source: Immun Ageing. 2024 May 17;21:32. doi: 10.1186/s12979-024-00435-2 (PMC11100119; doi:10.1186/s12979-024-00435-2)
Supplement: Supplementary file 1 — Supplementary Material 1. [file 12979_2024_435_MOESM1_ESM.docx]

### Supplemental material

Supplementary Table 1: Comparison of the ADNI Participants in the Lower and Upper Median NLR at Baseline

|  | Lower-Median | Upper-Median | p |  |
| --- | --- | --- | --- | --- |
| n | 55 | 55 |  |  |
| Age (Mean, SD) | 72.77 (6.63) | 74.97 (6.02) | 0.071 |  |
| Sex (m) (n, %) | 30 (54.5) | 25 (45.5) | 0.446 |  |
| Education (Mean, SD) | 16.49 (2.53) | 15.98 (2.94) | 0.333 |  |
| Race (white) (n, %) | 45 (81.8) | 49 (89.1) | 0.417 |  |
| APOE4 (n, %) | 18 (32.7) | 14 (25.5) | 0.529 |  |
| Days Between Exams (Mean, SD) | 79.58 (82.47) | 84.73 (114.67) | 0.788 |  |
| BMI (Mean, SD) | 27.91 (4.03) | 27.82 (5.16) | 0.920 |  |
| History of Hypertension (n, %) | 22 (40.0) | 30 (54.5) | 0.181 |  |
| Diabetic (n, %) | 1 (1.8) | 1 (1.8) | 1.000 |  |
| Cognitive Decline (n, %) | 1 (1.8) | 10 (18.2) | 0.011 | * |
| CSF Aβ Positive (n, %) | 12 (21.8) | 26 (47.3) | 0.009 | ** |
| CSF Aβ (Mean, SD) | 220 (49) | 202 (60) | 0.098 |  |
| CSF T-Tau (Mean, SD) | 65 (36) | 73 (29) | 0.231 |  |
| CSF P-Tau (Mean, SD) | 30 (23) | 31 (18) | 0.902 |  |
| NLR (Mean, SD) | 1.48 (0.31) | 2.97 (0.86) | <0.001 | *** |

Categorical variable differences were calculated using chi-squared tests, and continuous variable differences were calculated using ANOVA tests. See Methods for variable definitions.

Supplementary Table 2: Comparison of the NYU Participants in the Lower and Upper Median NLR at Baseline

|  | Lower-Median | Upper-Median | p |  |
| --- | --- | --- | --- | --- |
| n | 95 | 95 |  |  |
| Age (Mean, SD) | 60.50 (11.53) | 62.55 (10.28) | 0.197 |  |
| Sex (m) (n, %) | 28 (29.5) | 42 (44.2) | 0.051 |  |
| Education (Mean, SD) | 16.65 (2.09) | 16.84 (2.19) | 0.543 |  |
| Race (white) (n, %) | 84 (88.4) | 90 (94.7) | 0.191 |  |
| APOE4 (n, %) | 27 (28.4) | 31 (32.6) | 0.636 |  |
| Days Between Exams (Mean, SD) | 25.52 (73.12) | 31.14 (71.00) | 0.592 |  |
| BMI (Mean, SD) | 25.80 (4.47) | 25.77 (4.34) | 0.965 |  |
| History of Hypertension (n, %) | 16 (16.8) | 15 (15.8) | 1.000 |  |
| Diabetic (n, %) | 1 (1.1) | 4 (4.2) | 0.365 |  |
| Cognitive Decline (n, %) | 4 (4.2) | 4 (4.2) | 1.000 |  |
| CSF Aβ Positive (n, %) | 20 (21.1) | 18 (18.9) | 0.856 |  |
| CSF Aβ (Mean, SD) | 705 (217) | 723 (246) | 0.591 |  |
| CSF T-Tau (Mean, SD) | 244 (105) | 305 (173) | 0.004 | ** |
| CSF P-Tau (Mean, SD) | 42 (13) | 49 (20) | 0.002 | ** |
| NLR (Mean, SD) | 1.46 (0.30) | 2.72 (0.79) | <0.001 | *** |

Categorical variable differences were calculated using chi-squared tests, and continuous variable differences were calculated using ANOVA tests. See Methods for variable definitions.

Supplementary Table 3: Associations between the Baseline NLR and Longitudinal CSF Outcomes in the ADNI and NYU Cohorts

|  | | Univariate Model | | Adjusted Model | |
| --- | --- | --- | --- | --- | --- |
| Cohort | CSF Outcome | NLR β | NLR p-value | NLR β | NLR p-value |
| **ADNI** | **Aβ42** | -12.596 | **0.020** | -12.980 | **0.014** |
|  | **t-tau** | 4.640 | 0.151 | 3.906 | 0.240 |
|  | **p-tau** | 2.322 | 0.247 | 2.565 | 0.208 |
| **NYU** | **Aβ42** | 24.765 | 0.184 | 20.046 | 0.281 |
|  | **t-tau** | 44.321 | **<0.001** | 26.525 | **0.017** |
|  | **p-tau** | 5.382 | **<0.001** | 3.594 | **0.012** |

Unadjusted and adjusted linear mixed regression models for the association of the NLR and the three outcome variables are shown (Aβ42, t-tau, p-tau). Adjusted models included age, sex, education, race, APOE4, time between CBC and CSF exams, BMI, history of hypertension, diabetes, and onset of cognitive decline throughout follow-up CSF collection. β and p-values are shown.

Supplementary Table 4: Associations between the Baseline NLR and Longitudinal CSF Outcomes in the Aβ Positive and Aβ Negative Subsets of the ADNI and NYU Cohorts

| **ADNI Cohort** | Aβ Negative (n=178) | | | | Aβ Positive (n=96) | | | |
| --- | --- | --- | --- | --- | --- | --- | --- | --- |
|  | Univariate Model | | Adjusted Model | | Univariate Model | | Adjusted Model | |
| CSF Outcome | β | p-value | β | p-value | β | p-value | β | p-value |
| Aβ42 | -2.450 | 0.560 | -4.302 | 0.343 | -1.858 | 0.689 | 2.405 | 0.651 |
| t-tau | 4.685 | 0.173 | 4.989 | 0.182 | 0.588 | 0.930 | -3.300 | 0.707 |
| p-tau | -0.023 | 0.989 | 0.490 | 0.789 | 2.881 | 0.537 | 0.506 | 0.933 |
| **NYU Cohort** | Aβ Negative (n=277) | | | | Aβ Positive (n=69) | | | |
|  | Univariate Model | | Adjusted Model | | Univariate Model | | Adjusted Model | |
| CSF Outcome | β | p-value | β | p-value | β | p-value | β | p-value |
| Aβ42 | 13.788 | 0.418 | 4.392 | 0.797 | 10.355 | 0.707 | 34.259 | 0.298 |
| t-tau | 20.912 | **0.045** | 12.157 | 0.230 | 171.210 | **<0.001** | 110.306 | **0.020** |
| p-tau | 3.433 | **0.014** | 2.634 | 0.061 | 15.456 | **0.002** | 8.410 | 0.130 |

Unadjusted and adjusted linear mixed regression models for the association of the NLR and the three outcome variables are shown (Aβ42, t-tau, p-tau). Adjusted models included age, sex, education, race, APOE4, time between CBC and CSF exams, BMI, history of hypertension, diabetes, and onset of cognitive decline throughout follow-up CSF collection. Aβ positivity was defined by the CSF cut-off threshold for each cohort. β and p-values are shown.

Supplementary Table 5: Meta Analysis of Associations between the Baseline NLR and CSF Outcomes in the ADNI and NYU Cohorts

| **Baseline CSF Measures** | Full Cohort (n=301) | | Aβ Negative (n=225) | | Aβ Positive (n=76) | |
| --- | --- | --- | --- | --- | --- | --- |
| CSF Outcome | Univariate Model | Adjusted Model | Univariate Model | Adjusted Model | Univariate Model | Adjusted Model |
| Aβ42 | NA | NA | p=0.697 | NA | NA | p=0.621 |
| t-tau | **p<0.001** | **p=0.016** | **p=0.014** | p=0.130 | NA | NA |
| p-tau | **p<0.001** | **p=0.012** | NA | P=0.247 | **p=0.029** | NA |
| **Longitudinal CSF Measures** | Full Cohort (n=620) | | Aβ Negative (n=455) | | Aβ Positive (n=165) | |
| CSF Outcome | Univariate Model | Adjusted Model | Univariate Model | Adjusted Model | Univariate Model | Adjusted Model |
| Aβ42 | NA | NA | NA | NA | NA | p=0.502 |
| t-tau | **p<0.001** | **p=0.018** | **p=0.027** | p=0.133 | p=0.148 | NA |
| p-tau | **p=0.001** | **p=0.011** | NA | p=0.194 | p=0.051 | p=0.712 |

Unadjusted and adjusted linear regression models for the association of the NLR and the three outcome variables are shown (Aβ42, t-tau, p-tau). Adjusted models included age, sex, education, race, APOE4, time between CBC and CSF exams, BMI, history of hypertension, and diabetes. In the longitudinal CSF analysis, adjusted models also included the onset of cognitive decline throughout follow-up CSF collection. GLM were used for baseline CSF associations, while LMM were used to incorporate follow-up CSF measures in the outcome. Aβ positivity was defined by the CSF cut-off threshold for each cohort. P-values are shown, except in instances where p-values could not be calculated since β coefficients were in opposite directions between cohorts.
